# Infectious etiology of intussusception in Indian children less than 2 years old: a matched case-control analysis

**DOI:** 10.1186/s13099-024-00659-z

**Published:** 2024-10-23

**Authors:** Ira Praharaj, Samarasimha Nusi Reddy, Nayana Prabhakaran Nair, Jacqueline Elizabeth Tate, Sidhartha Giri, Varunkumar Thiyagarajan, Venkata Raghava Mohan, Rajendiran Revathi, Kalaivanan Maheshwari, Priya Hemavathy, Nirmal Kumar, Mohan Digambar Gupte, Rashmi Arora, Sowmiya Senthamizh, Suhasini Mekala, Krishna Babu Goru, Padmalatha Pamu, Manohar Badur, Subal Pradhan, Mrutunjay Dash, Nirmal Kumar Mohakud, Rajib Kumar Ray, Geetha Gathwala, Madhu Gupta, Ravi Kanojia, Rajkumar Gupta, Suresh Goyal, Pramod Sharma, Mannancheril Abraham Mathew, Tarun John Kochukaleekal Jacob, Balasubramanian Sundaram, Chethrapilly Purusothaman Girish Kumar, Priyadarshini Dorairaj, Ramasubramaniam Pitchumani, Raghul Maniam, Sambandan Kumaravel, Hemant Jain, Jayanta Kumar Goswami, Ashish Wakhlu, Vineeta Gupta, Jie Liu, Eric R. Houpt, Umesh D. Parashar, Gagandeep Kang

**Affiliations:** 1 The Wellcome Trust Research Laboratory, Division of Gastrointestinal Sciences, Christian Medical College, Vellore, Tamil Nadu India; 2 ICMR Regional Medical Research Centre, Bhubaneswar, Odisha India; 3 Centers for Disease Control and Prevention, Atlanta, GA USA; 4 Department of Community Health, Christian Medical College, Vellore, Tamil Nadu India; 5 Translational Health Science and Technology Institute, Faridabad, India; 6 Kurnool Medical College and Government General Hospital, Kurnool, Andhra Pradesh India; 7 Government General Hospital and Rangaraya Medical College, Kakinada, Andhra Pradesh India; 8 King George Hospital and Andhra Medical College, Visakhapatnam, Andhra Pradesh India; 9 Sri Venkateshwara Medical College, Tirupati, Andhra Pradesh India; 10 Sardar Vallabh Bhai Patel Post Graduate Institute of Paediatrics, Cuttack, Odisha India; 11 Institute of Medical Sciences, SUM Hospital, Bhubaneswar, Odisha India; 12 Kalinga Institute of Medical Sciences, Bhubaneswar, Odisha India; 13 Hi-Tech Hospital, Bhubaneswar, Odisha India; 14 Pandit Bhagwat Dayal Sharma Post Graduate Institute of Medical Sciences, Rohtak, Haryana India; 15 Post Graduate Institute of Medical Education and Research, Chandigarh, India; 16 Sawai Man Singh Medical College, Jaipur, Rajasthan India; 17 Rabindranath Tagore Medical College, Udaipur, Rajasthan India; 18 Dr. Sampurnanand Medical College, Jodhpur, Rajasthan India; 19 Malankara Orthodox Syrian Church Medical College Hospital, Kolencherry, Kerala India; 20 Department of Pediatric Surgery, Christian Medical College, Vellore, Tamil Nadu India; 21 Kanchi Kamakoti Child Trust Hospital, Chennai, Tamil Nadu India; 22 National Institute of Epidemiology, Chennai, Tamil Nadu India; 23 Institute of Child Health, Chennai, Tamil Nadu India; 24 Government Rajaji Hospital and Madurai Medical College, Madurai, Tamil Nadu India; 25 Coimbatore Medical College, Coimbatore, Tamil Nadu India; 26 Jawaharlal Nehru Institute of Post-graduate Medical Education & Research, Puducherry, India; 27 Mahatma Gandhi Memorial Medical College, Indore, Madhya Pradesh India; 28 Government Medical College, Guwahati, Assam India; 29 King George Medical College, Lucknow, Uttar Pradesh India; 30 Institute of Medical Sciences, Banaras Hindu University, Varanasi, Uttar Pradesh India; 31 Division of Infectious Diseases and International Health, University of Virginia, Charlottesville, USA

**Keywords:** Intussusception, Case-control, Adenovirus, Viral pathogens, PAF

## Abstract

**Background:**

Enteric infections are hypothesized to be associated with intussusception in children. A small increase in intussusception following rotavirus vaccination has been seen in some settings. We conducted post-marketing surveillance for intussusception following rotavirus vaccine, Rotavac introduction in India and evaluated association of intussusception with enteric pathogens.

**Methods:**

In a case-control study nested within a large sentinel hospital-based surveillance program in India, stool samples from 272 children aged less than 2 years admitted for intussusception and 272 age-, gender- and location-matched controls were evaluated with Taqman array card based molecular assays to detect enteric viruses, bacterial enteropathogens and parasites. Matched case-control analysis with conditional logistic regression evaluated association of enteropathogens with intussusception. Population attributable fractions (PAF) were calculated for enteropathogens significantly associated with intussusception.

**Results:**

The most prevalent enteropathogens in cases and controls were enteroaggregative *Escherichia coli*, adenovirus 40/41, adenovirus C serotypes and enteroviruses. Children with intussusception were more likely to harbor adenovirus C serotypes (adjusted odds-ratio (aOR) = 1.74; 95% confidence interval (CI) 1.06–2.87) and enteroviruses (aOR = 1.77; 95% CI 1.05–2.97) than controls. Rotavirus was not associated with increased intussusception risk. Adenovirus C (PAF = 16.9%; 95% CI 4.7% − 27.6%) and enteroviruses (PAF = 14.7%; 95% CI 4.2% − 24.1%) had the highest population attributable fraction for intussusception.

**Conclusion:**

Adenovirus C serotypes and enteroviruses were significantly associated with intussusception in Indian children. Rotavirus was not associated with risk of intussusception.

**Supplementary Information:**

The online version contains supplementary material available at 10.1186/s13099-024-00659-z.

## Background

Intussusception, the invagination of one segment of the intestine into a segment of the distal intestine, is the most common cause of acute intestinal obstruction in infants, with peak incidence in the age group of 4–6/10 months [1, 2]. The reported baseline rates and median age of intussusception vary between countries and by geography [3]. It is unclear if these wide regional differences are due to particular environmental risk factors such as food habits, infectious agents or due to genetic predisposition and ethnicity. In post-licensure evaluations of the additional risk of intussusception after rotavirus vaccination, a slightly higher risk has been found in developed and middle-income settings [4–6], but not in low-income settings in Africa [7, 8] and in India [9, 10] where the exposure to enteric pathogens is early and considerable. While infections are hypothesized to be associated with intussusception in infants since enteric viruses have been found in anatomical lead points of intussusception in some studies [11], and high prevalence of viruses has been reported in stool samples from cases of intussusception [12], there is paucity of comparative analyses of enteric pathogen profiles among intussusception cases and age-matched controls.

A recent case-control study among children < 2 years age in four Asian countries, Bangladesh, Pakistan, Nepal and Vietnam used molecular methods to evaluate infectious etiologies of intussusception in settings where rotavirus vaccine had not been introduced and reported adenovirus and human herpes virus-6 (HHV-6) as significantly associated with intussusception [13]. Our study has used sensitive molecular methods to study the infectious etiology in intussusception and to address the lack of data on infectious etiologies of intussusception among Indian infants.

## Methods

### Case and control selection

A prospective multi-centric hospital based active surveillance for intussusception in infants and children less than 2 years of age was conducted in 27 sites across 10 states of India from April 2016 to June 2019 [9, 14]. Detailed description of the surveillance and the primary analysis for studying association of vaccination with Rotavac, an indigenously manufactured vaccine that was licensed in 2014 and introduced into the national immunization program in 2016, and intussusception in infants is published elsewhere [9].

Infants and children less than 2 years of age and admitted to a study hospital for intussusception, with Brighton Level 1 of diagnostic certainty, were included as part of the active surveillance for intussusception. Brighton level 1 of diagnostic certainty includes surgical demonstration of invagination and/or specific radiologic criteria for intussusception demonstrated by air or liquid enema, or the presence of an intra-abdominal mass with intussusception specific features [1].

For a sub-set of the cases of intussusception recruited as part of the active surveillance for vaccine safety [9], age (+/- 42 days), sex and location (state of residence) matched controls were enrolled. Controls had a clinical diagnosis not related to the gastrointestinal tract. All infants and children satisfying these criteria were included irrespective of rotavirus vaccination status. Stool specimens were obtained from cases and controls within 48 h of enrolment and transported under appropriate cold chain to the laboratory where the samples were stored frozen until they were shipped to the designated central laboratory for molecular testing of enteropathogens.

### Sample size calculation

The estimated number of cases of intussusception and matched controls required to demonstrate a 10% difference in pathogen prevalence between cases and controls with 80% power was 140 cases and matched controls [14].

### Consent and ethical approval

The study was approved by the institutional review board (IRB) of Christian Medical College, Vellore (IRB :10692(OBSERVE) dated 21.06.2017) and institutional ethical committees of all participating sites. The list of participating sites and the number of matched case-control pairs included from each of these sites is included in supplementary table S1. All eligible cases and controls were enrolled only after written informed consent was obtained from their parents/guardians.

### Molecular detection of enteropathogens in stool specimens

Stool samples were collected from cases and matched controls within 48 h of enrolment and stored at -20̊ C till the samples were shipped to the designated testing laboratory – Wellcome Trust Research Laboratory, Division of Gastrointestinal Sciences, Christian Medical College, Vellore. In the testing laboratory, stool samples were stored at -80̊ C till testing.

Total nucleic acid was extracted from stool specimens using established methods [15]. Extracted nucleic acid was used to amplify DNA and RNA targets on TaqMan array card (TAC) assays. The targets on the Taqman array cards included pathogens hypothesized to have an association with intussusception based on available literature [13]. Gene targets specific for rotavirus vaccine strains for Rotarix, RotaTeq and Rotavac were also included. The list of enteropathogen targets and primers and probes for the different targets is available in supplementary table S2. We excluded the presence of Sabin vaccine strains among enterovirus target positive samples by performing multiplex qPCR for Sabin 1 and Sabin 3 [16].

### Statistical analysis

Cycle threshold (Ct) value of 35 cycles was used as the analytical cut off for all enteropathogens with targets on the TAC assays for the primary analysis with specimens with Ct value ≤ 35 cycles considered positive for a target. Since a number of studies have shown the strong correlation of Cq values for specific enteric virus targets with clinical outcomes and disease severity, we also performed a sensitivity analysis using (Cycle of quantitation) Cq values of 30 cycles and 25 cycles for different enteropathogen targets. Conditional logistic regression analysis was performed and odds ratios with 95% CI calculated for association of different enteropathogens with intussusception. Population attributable fraction (PAF) was calculated for all enteropathogens significantly associated with intussusception using PUNAFCC module in STATA 15 [17]. P values less than 0.05 were considered significant and all reported P values are two-sided. All analyses were performed using Stata 15 software (StataCorp. 2017. Stata Statistical Software: Release 15. College Station, TX: StataCorp LLC.) and GraphPad Prism 6 (GraphPad Software Inc., La Jolla, San Jose, CA, USA).

## Results

The analysis included a total of 272 matched case-control pairs from 23 sites participating in the intussusception surveillance across 10 states in India (Supplementary Table no. S1). Each control included in the analysis was matched for age (+/- 42 days), gender and the state of residence with a case of intussusception as per the inclusion criteria. 69.5% of intussusception cases included in this analysis were males. The most common clinical features were vomiting in 75% (204/272), fever in 32.7% (89/272) and diarrhea in 41.2% (112/272) of all cases. The commonest mode of management was surgical reduction in 59%(189/272) of cases followed by hydrostatic or pneumatic reduction in 29.8% and intestinal resection in 11.0% of cases(Supplementary Table S3). Vaccination history with documentation for one or more doses of oral rotavirus vaccines was available for 55.5% (151/272) of cases and 55.9% (152/272) of matched controls included in this analysis.

### Prevalence of enteropathogens in intussusception cases versus controls

**Fig. 1:**
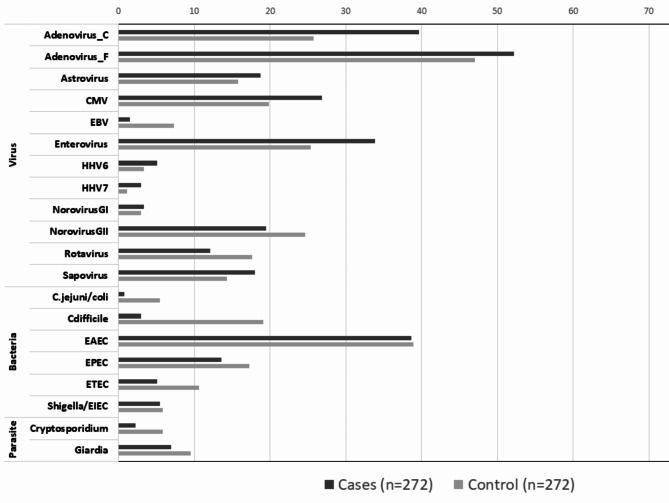
Prevalence of enteropathogens in intussusception cases and matched controls

The median number of enteropathogens detected in cases and controls was 3 (IQR = 2 for both cases and controls). Using a Cq value of 35 for the enteropathogen targets, the most prevalent enteropathogens were enteroaggregative *Escherichia coli* (EAEC) (38.6% in cases versus 39% in controls), Adenovirus 40/41 (52.2% in cases versus 47.1% in controls), adenovirus C serotypes (39.7% in cases versus 25.7% in controls) and enteroviruses (33.8% in cases versus 25.4% in controls). Figure 1 presents the prevalence of enteropathogens including enteric viruses, enteropathogenic bacteria and parasites among intussusception cases and matched controls.

### Matched case-control analysis and population attributable fraction

Matched odds-ratios were calculated for each major enteropathogen target included in the testing and its association with intussusception by conditional logistic regression analysis. Cases of intussusception were significantly more likely to harbor adenovirus C serotypes (matched odds ratio = 1.74 95% CI 1.06–2.87) compared to controls, while the association between adenovirus 40/41 with intussusception was not statistically significant using a Cq of 35 cycles. The association between detection of adenovirus C serotypes and intussusception was stronger when lower Cq values (higher pathogen load) were used – Matched odds-ratio = 2. 46 (95% CI 1.36–5.04) at Cq 30 cycles; matched odds-ratio = 6.91 (95% CI 1.56–30.68) at Cq 25 cycles (Table S4 & Table S5).

**Fig. 2:**
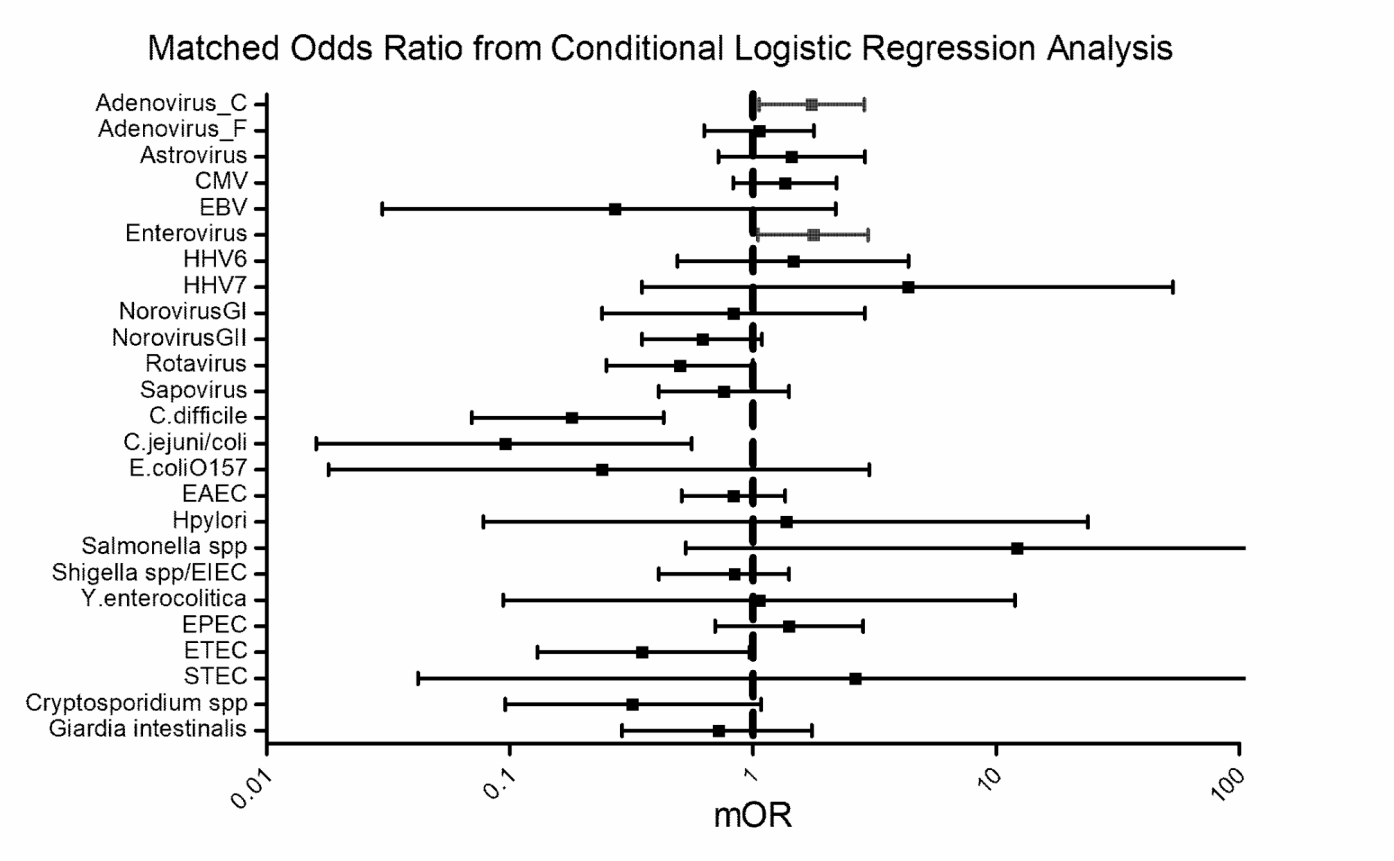
Enteropathogen presence and matched odds ratio (± 95% CI) of intussusception (Cq 35 cycles)

**Fig. 3:**
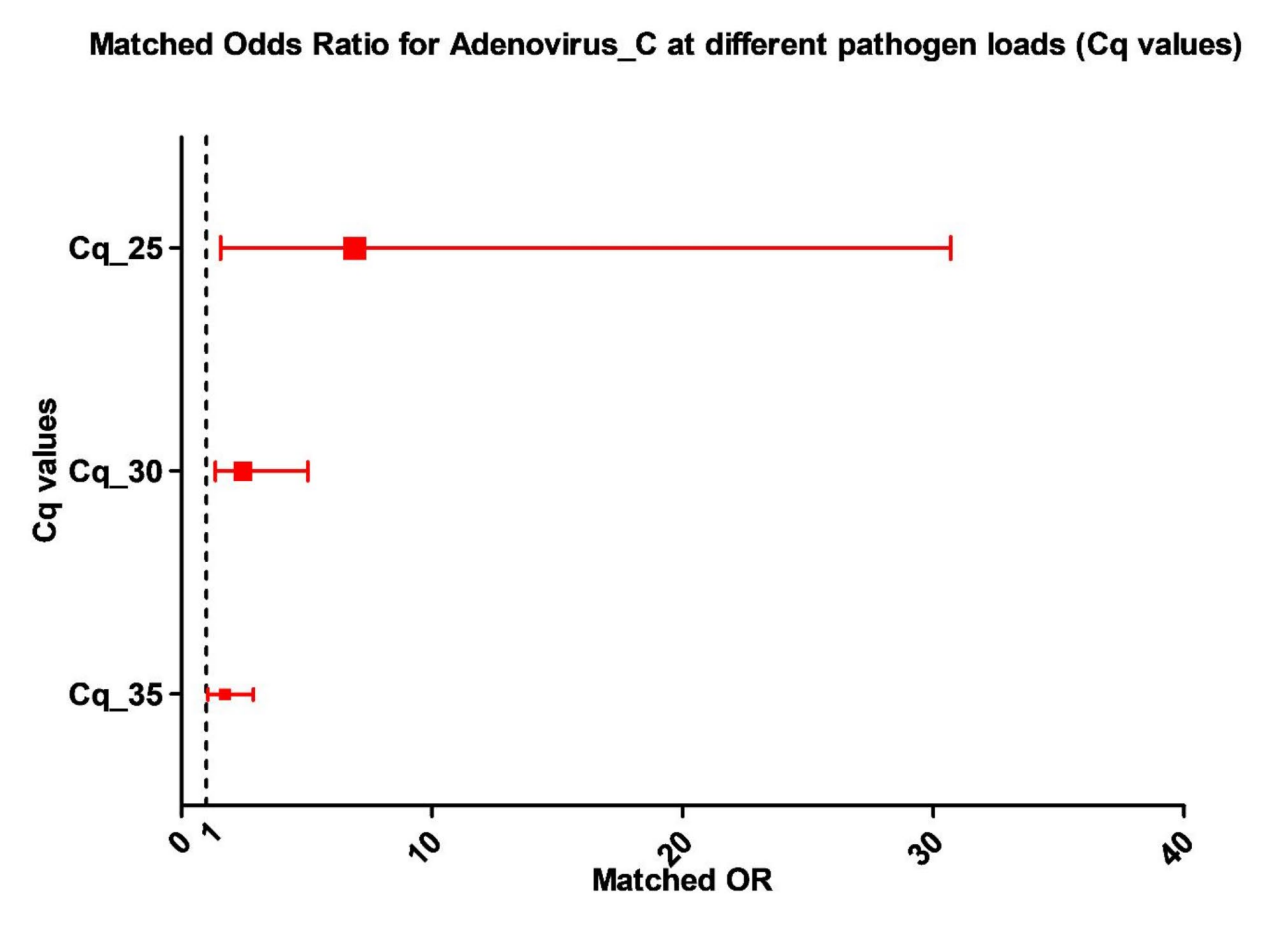
Adenovirus C quantity (Cq values) and matched odds ratio (± 95% CI) for intussusception

Figure 2 shows the matched odds-ratio for intussusception for the different enteropathogens while Fig. 3 shows the increasing magnitude of association of adenovirus C serotype infection with intussusception at higher viral load (lower Cq values).

Enterovirus detection at Cq of 35 cycles and 30 cycles was also significantly associated with intussusception (At Cq of 35 cycles, odds-ratio = 1.77; 95% CI 1.05–2.97; at Cq of 30 cycles, odds-ratio = 1.76; 95% CI 0.93–3.3). We excluded the presence of Sabin vaccine strains among enterovirus target positive samples by performing multiplex qPCR for Sabin 1 and Sabin 3 (data not shown). Non-polio enterovirus (NPEV) was detected in 30.5% (83/272) of intussusception cases versus 24.3% (66/272) of controls (Odds ratio = 1.37; 95% CI 0.92–2.04).

Detection of rotavirus target was not associated with increased risk intussusception at Cq of 35 (matched OR: 0.5 95% CI 0.25 -1) and 30 cycles (matched OR: 0.38 95% CI 0.15–0.93). 33 out of 272 cases tested were positive for the rotavirus target at a Ct value cut-off of 35 cycles, while 48 out of 272 controls were positive for the rotavirus target. We performed genotyping to characterize rotaviruses for all specimens where the pan-rotavirus target was detected by TAC. In addition, the TAC assays included targets from the rotavirus vaccine strains of Rotavac, Rotarix and RotaTeq. Rotavirus genotypes could be ascertained in only 9/33 of the samples from intussusception cases positive for rotavirus target. The commonest rotavirus genotype detected among cases positive for rotavirus was G3P [8] (6/33), followed by G1P [6] (3/33). Among controls positive for the rotavirus target, G3P [8] was detected in 8/48 samples, G2P [4] in 6/48 and G1P [8] in one sample. None of the oral rotavirus vaccine strains for which targets were included in the TAC assays (Rotarix, RotaTeq and Rotavac) were detected in any of the samples from cases or controls.

*Clostridium difficile* detection was more common among controls compared to intussusception cases (19.1% among controls versus 2.9% among cases at Cq value of 35 cycles). Presence of *C. difficile* was significantly protective against intussusception, irrespective of the Cq values used as the cut-off for target positivity (matched odds-ratio at Cq of 35 cycles = 0.18; 95% CI 0.07–0.43). Co-infection with more than one enteropathogen was detected in 76.4% (208/272) of all cases and 79.8% (217/272) of matched controls.

### Population Attributable Fraction (PAF)

Table 1. presents the matched odds-ratio and PAF for the different enteropathogens which were detected using TAC assays in this study. Enteropathogens with the highest population attributable fractions (PAF) for intussusception were adenovirus C serotypes (PAF = 16.9%; 95% CI – 4.7 − 27.5%) and enteroviruses (PAF = 14.7%; 95% CI – 4.2 − 24.1%).

**Table 1:** Matched odds ratios and PAF for different enteropathogens (cq of 35 cycles)

Pathogen	Positivity in cases *N*(%)	Positivity in controls *N*(%)	OR	95% CI		*P*>|z|	PAF	95%CI	
Adenovirus C	108 (39.71)	70 (25.74)	1.7	1.1	2.9	0.029	16.9	4.7	27.5
Adenovirus F (40/41)	142 (52.21)	128 (47.06)	1.0	0.6	1.8	0.825			
Astrovirus	51 (18.75)	43 (15.81)	1.4	0.7	2.9	0.298			
CMV	73 (26.84)	54 (19.85)	1.3	0.8	2.2	0.218			
EBV	4 (1.47)	20 (7.35)	0.2	0.0	2.2	0.221			
Enterovirus	92 (33.82)	69 (25.37)	1.7	1.1	3.0	0.031	14.7	4.2	24.1
HHV6	14 (5.15)	9 (3.31)	1.4	0.5	4.4	0.483			
HHV7	8 (2.94)	3 (1.10)	4.3	0.4	53.3	0.251			
Norovirus GI	9 (3.31)	8 (2.94)	0.8	0.2	2.9	0.772			
Norovirus GII	53 (19.49)	67 (24.63)	0.6	0.4	1.1	0.101			
Rotavirus	33 (12.13)	48 (17.65)	0.5	0.3	1	0.051			
Sapovirus	49 (18.01)	39 (14.34)	0.8	0.4	1.4	0.384			
*Clostridium difficile*	2 (0.74)	15 (5.51)	0.18	0.1	0.4	< 0.001			
*Campylobacter jejuni/ coli*	8 (2.94)	52 (19.12)	0.096	0.0	0.6	0.009			
*E.coli* O157	1 (0.37)	2 (0.74)	0.2	0.0	3.0	0.266			
EAEC	105 (38.60)	106 (38.97)	0.8	0.5	1.4	0.463			
*Helicobacter pylori*	4 (1.47)	2 (0.74)	1.4	0.1	23.9	0.83			
*Salmonella* spp	4 (1.47)	2 (0.74)	12.2	0.5	281.2	0.119			
*Shigella*/EIEC	15 (5.51)	16 (5.88)	0.9	0.4	1.4	0.384			
EPEC	37 (13.6)	47 (17.28)	1.4	0.7	2.8	0.332			
ETEC	14 (5.15)	29 (10.66)	0.4	0.13	1.0	0.043			
STEC	2 (0.74)	1 (0.37)	2.6	0.0	165.0	0.647			
*Cryptosporidium* spp	6 (2.21)	16 (5.88)	0.3	0.1	1.1	0.067			
*Giardia intestinalis*	19 (6.99)	26 (9.56)	0.7	0.3	1.8	0.463			

### Enteropathogens in samples from cases of intussusception and controls within the 21-day risk-window after rotavirus vaccination

In the primary self-controlled case series (SCCS) analysis for assessing risk of intussusception due to rotavirus vaccination, a 21-day window after the rotavirus vaccine dose had been considered as the risk window [9]. The detailed methods and primary analysis have been reported previously [9, 14]. For 18 cases of intussusception from whom stool samples were included in the enteropathogen analysis, the intussusception episode had occurred within 21 days of a rotavirus vaccine dose. Adenovirus C serotypes were detected in 10 of the cases, while adenovirus 40/41 was detected in 11. Pan- rotavirus target, NSP3 was detected in only 3 cases while the Rotavac vaccine strain or any other rotavirus vaccine strain was not detected in any of these cases.

For 21 controls included in this analysis, stool samples had been collected within 21 days of rotavirus vaccine dose. Adenovirus C serotypes were detected in only 5 controls, adenovirus 40/41 in 11. While wild-type rotavirus was detected in 3 controls, none of the rotavirus vaccine strains were detected in any samples from controls. Enteropathogen positivity among samples from intussusception cases and controls obtained during the 21 day “risk period” of a rotavirus vaccine dose is presented in Table S6.

## Discussion

Using sensitive molecular tools for the detection of enteropathogens in 272 cases of intussusception and matched controls, we show an association of adenoviruses belonging to species C with intussusception among Indian children less than 2 years of age. Our findings are consistent with earlier reports from Vietnam and Australia [12]. Although previously suggested [18, 19], it is only recently that comprehensive analyses from case-control studies have shown convincing evidence of adenovirus serotypes with intussusception [13]. Adenoviruses are a diverse group of viruses with 88 serotypes classified into 7 different species, A to G [20], associated with clinical conditions ranging from diarrhea, respiratory manifestations to particularly serious disease among the immunosuppressed and immunocompromised population [21]. Species C adenoviruses are among the commonest species reported from clinical infections and are described as respiratory pathogens. Earlier studies have suggested association of respiratory and non-enteric adenoviruses with intussusception [12, 19, 22]. The association between the respiratory tissue tropic adenovirus C serotypes and intussusception may appear counter- intuitive. However, this is consistent with clinical reports of respiratory symptoms preceding intussusception [12, 23, 24]. Presence of adenovirus C serotypes in immunohistochemical analysis of necrotic tissues from intussusception cases has been demonstrated [24]. It is hypothesized that the host immune response, rather than the presence of adenovirus C serotypes themselves, might be responsible for the association of these pathogens with intussusception [18]. In addition, adenovirus C serotypes are typically associated with latency in mucosa-associated lymphoid tissue (MALT), particularly within the T-cell population of the tonsils and long-term intermittent excretion of these viruses in fecal samples has been reported [20].

Enteroviruses were also significantly associated with intussusception in our study. Similar findings have been reported in a study from western Australia specifically for enterovirus B [25]. However, a multi-centre study across 4 Asian countries did not find a significant association of enterovirus infections with intussusception [13]. In our study, we distinguished between NPEV and Sabin vaccine serotypes but did not characterize the serotypes of all the NPEV infections.

We did not find any association between rotavirus detection in stool specimens and intussusception. Our findings are consistent with earlier studies where no association between wild-type rotavirus and intussusception was found [12, 13, 26]. This includes the findings from the Asian intussusception surveillance network. We were able to distinguish between wild-type rotavirus and vaccine strains since genotyping for VP4 and VP7 genes was performed for all pan-rotavirus positives and targets from rotavirus vaccine strains of Rotavac, Rotarix and RotaTeq were included in the TAC assays. The lack of finding of any vaccine rotavirus strain further supports lack of association between the vaccine and intussusception in Indian infants [9].

Detection of *C. difficile* was protective against intussusception in infants. Asymptomatic C. difficile carriage is common among infants and children less than 2 years of age and prospective screening for *C. difficile* in infants and children less than 2 years has revealed colonization rates of around 33% overall, higher than in adults [27]. As much as 25% of all C. difficile positive children in hospital settings have been found to be asymptomatic [28]. Mechanisms suggested for the apparent protection of infants against *C. difficile* disease despite colonization include lack of toxin receptors on intestinal cells in infants, role of breast milk as a protective factor and protection due to other intestinal flora among infants [29].

Unlike the reported findings from a recent study from 4 Asian countries [13], detection of HHV-6 was not significantly associated with intussusception in this study and the absolute number of intussusception cases and controls in whom HHV-6 was detected were also low (14 cases and 9 controls).

This is the first comprehensive study from India evaluating infectious etiologies of intussusception in children less than 2 years of age. The Asian Intussusception Surveillance Network study which used methods similar to our study for describing the infectious etiology of intussusception in 4 Asian countries did not have any study sites from India [13]. Since our study involved 23 sentinel sites across 10 states of India, much of the findings of this study can be generalized to rest of India.

With the inclusion of 272 matched case-control pairs, the study had 90% power to detect enteropathogen prevalence difference of 10% between cases and controls at a significance level of 5%.

This study had limitations. The presence of enteropathogens was detected in stool samples and not directly from intestinal biopsy specimens. While the study showed that presence of adenovirus is associated with intussusception in Indian settings as in other Asian countries, this does not explain the considerable difference in incidence of intussusception in different regions, e.g. Vietnam and China having considerably higher incidence of intussusception compared to other regions of the world [13, 30]. While the associations between intussusception and Adenovirus C and enteroviruses were found to be statistically significant in the matched case-control analysis reported here, these infectious etiologies probably represent a small fraction of the possible causes of intussusception. It is also important to generate more data for other parts of the world before extrapolating our study findings to other geographical regions and socioeconomic settings where the exposure to enteric pathogens and the profile of the infant gut microbiome might be considerably different.

## Conclusions

In conclusion, this study presents evidence of association of adenovirus C and enterovirus infections and intussusception among Indian infants and children less than 2 years of age. The findings of the study also provide further support to the lack of association of current vaccine strains of oral rotavirus vaccines (ORV) being used in Indian settings with intussusception in infants and hence to the evidence base for the safety of these licensed vaccines.

## Electronic supplementary material

Below is the link to the electronic supplementary material.

Supplementary Material 1

Supplementary Material 2

Supplementary Material 3

Supplementary Material 4

Supplementary Material 5

Supplementary Material 6

## Data Availability

Additional data supporting findings reported in the manuscript are available as supplementary data. Any additional information or data pertaining to this manuscript and results will be made available on reasonable request.

## Abbreviations

TAC: TaqMan array card
C. difficile: Clostridium difficile
Ct: Cycle threshold (Ct)
Cq: Cycle of quantitation
HHV-6: Human Herpes Virus-6
OR: Odds Ratio
PAF: Population attributable fraction
