# Supplementary material for: Infectious etiology of intussusception in Indian children less than 2 years old: a matched case-control analysis
Source: Gut Pathog. 2024 Oct 23;16:61. doi: 10.1186/s13099-024-00659-z (PMC11515542; doi:10.1186/s13099-024-00659-z)
Supplement: Supplementary file 1 — Supplementary Material 1 [file 13099_2024_659_MOESM1_ESM.docx]

| **State** | **City** | **Hospital name** | **No. of intussusception**  **case-control pairs included** |
| --- | --- | --- | --- |
| Andhra Pradesh | Kurnool | Kurnool Medical College | 1 |
| Andhra Pradesh | Kakinada | Government General Hospital and Rangaraya Medical College | 7 |
| Andhra Pradesh | Vishakhapatnam | King George Hospital and Andhra Medical College | 7 |
| Andhra Pradesh | Tirupati | Sri Venkateshwara Medical College | 7 |
| Assam | Guwahati | Gauhati Medical College | 3 |
| Haryana | Rohtak | Pandit Bhagwat Dayal Sharma Post Graduate Institute of Medical Sciences | 8 |
| Chandigarh | Chandigarh | Post Graduate Institute of Medical Education and Research | 70 |
| Madhya Pradesh | Indore | Mahatma Gandhi Memorial Medical College | 11 |
| Odisha | Cuttack | Sardar Valla Bhai Patel Post Graduate Institute of Paediatrics | 35 |
| Odisha | Bhubaneswar | Institute of Medical Sciences and SUM Hospital | 5 |
| Odisha | Bhubaneswar | Kalinga Institute of Medical Sciences | 2 |
| Odisha | Bhubaneswar | Hi-Tech Hospital | 1 |
| Puducherry | Puducherry | Jawaharlal Nehru Institute of Post-graduate Medical Education & Research (JIPMER) | 9 |
| Rajasthan | Jaipur | Sawai Man Singh Medical College | 30 |
| Rajasthan | Udaipur | Rabindranath Tagore Medical College | 8 |
| Rajasthan | Jodhpur | Dr. Sampurnanand Medical College | 19 |
| Tamil Nadu | Vellore | Christian Medical College | 6 |
| Tamil Nadu | Vellore | Government Vellore Medical College | 1 |
| Tamil Nadu | Chennai | Kanchi Kama Koti Child Trust Hospital | 16 |
| Tamil Nadu | Chennai | Institute of Child Health | 15 |
| Tamil Nadu | Madurai | Government Rajaji Hospital and Madurai Medical College | 6 |
| Uttar Pradesh | Lucknow | King George Medical College | 4 |
| Uttar Pradesh | Varanasi | Institute of Medical Sciences, Banaras Hindu University | 1 |
