# Supplementary material for: Infectious etiology of intussusception in Indian children less than 2 years old: a matched case-control analysis
Source: Gut Pathog. 2024 Oct 23;16:61. doi: 10.1186/s13099-024-00659-z (PMC11515542; doi:10.1186/s13099-024-00659-z)
Supplement: Supplementary file 2 — Supplementary Material 2 [file 13099_2024_659_MOESM2_ESM.docx]

| **Enteropathogen target** | **Forward primer 1** | **Forward primer 2** | **Reverse primer 1** | **Reverse primer 2** | **Probe 1** | **Probe 2** | **Reference** |
| --- | --- | --- | --- | --- | --- | --- | --- |
| Adenovirus_C | GGACCGCATGTACTCSTTCTT |  | TTTAGTATCRTCCACCACCTGACG | TTTAGTATCGTCCACCACTTGCC | AGAAACTTCCAGCCCATGAGC |  | Liu J, 2016 |
| Adenovirus_F | AACTTTCTCTCTTAATAGACGCC |  | AGGGGGCTAGAAAACAAAA |  | CTGACACGGGCACTCT |  |  |
| Adenovirus_pan | GCCACGGTGGGGTTTCTAAACTT |  | GCCCCAGTGGTCTTACATGCACATC |  | TGCACCAGACCCGGGCTCAG |  |  |
| Ancylostoma | GAATGACAGCAAACTCGTTGTTG |  | ATACTAGCCACTGCCGAAACGT |  | ATCGTTTACCGACTTTAG |  |  |
| Ascaris | GCCACATAGTAAATTGCACACAAAT |  | GCCTTTCTAACAAGCCCAACAT |  | TTGGCGGACAATTGCATGCGAT |  |  |
| Astrovirus | CAGTTGCTTGCTGCGTTCA |  | CTTGCTAGCCATCACACTTCT |  | CACAGAAGAGCAACTCCATCGC |  |  |
| C_difficile | GGTATTACCTAATGCTCCAAATAG | TTCAAGCAGAAATAGAGCACTC | TTTGTGCCATCATTTTCTAAGC | TATCAGCCCATTGTTTTATGTATTC | CCTGGTGTCCATCCTGTTTC | CACTGACTTCTCCACCTATCCA |  |
| C_jejuni-coli | CTGCTAAACCATAGAAATAAAATTTCTCAC |  | CTTTGAAGGTAATTTAGATATGGATAATCG | | CATTTTGACGATTTTTGGCTTGA |  |  |
| Campy_Pan | AAAGTIGGMAAAGATGGTGTTAT | AAAGTIGGWAAAGACGGYGTTAT | TCAAATTGCATACCYTCAAC |  | TTTGCCTCTTCMACAGT | TTTGCTTCTTCWACAGT |  |
| CMV | AGG TCTTCA AGG AAC TCA GCA AGA | GAG CCC GACTTT ACC ATC CA | CGG CAA TCG GTT TGT TGT AAA | CAG CCG GCG GTA TCG A | AMC CCG TCA GCC ATT CTCTCG GC | ACC GCA ACA AGA TT |  |
| Cryptosporidium | GGGTTGTATTTATTAGATAAAGAACCA |  | AGGCCAATACCCTACCGTCT |  | TGACATATCATTCAAGTTTCTGAC |  |  |
| E_histolytica | ATTGTCGTGGCATCCTAACTCA |  | GCGGACGGCTCATTATAACA |  | TCATTGAATGAATTGGCCATTT |  |  |
| EAEC_aggR | GCAATCAGATTAARCAGCGATACA |  | TTCGGACAACTRCAAGCATC |  | AAGACGCCTAAAGGATGCCC |  |  |
| EBV | CGGAAGCCCTCTRGACTTC |  | CCCTGTTTATCCGATGGAATG |  | TGTACACGCACGAGAAATGCG |  |  |
| Enterovirus | CCCTGAATGCGGCTAATCC |  | GCGATTGTCACCATWAGCAG |  | CCGACTACTTTGGGWGTCCGT |  |  |
| EPEC_bfpA | TGGTGCTTGCGCTTGCT |  | CGTTGCGCTCATTACTTCTG |  | CAGTCTGCGTCTGATTCCAA |  |  |
| EPEC_eae | CATTGATCAGGATTTTTCTGGTGATA |  | CTCATGCGGAAATAGCCGTTA |  | ATACTGGCGAGACTATTTCAA |  |  |
| ETEC_LT | TTCCCACCGGATCACCAA |  | CAACCTTGTGGTGCATGATGA |  | CTTGGAGAGAAGAACCCT |  |  |
| ETEC_ST | GCTAAACCAGYAGRGTCTTCAAAA | TGAATCACTTGACTCTTCAAAA | CCCGGTACARGCAGGATTACAACA | GGCAGGATTACAACAAAGTT | TGGTCCTGAAAGCATGAA | TGAACAACACATTTTACTGCT |  |
| Giardia | GACGGCTCAGGACAACGGTT |  | TTGCCAGCGGTGTCCG |  | CCCGCGGCGGTCCCTGCTAG |  |  |
| H_pylori | GACACCAGAAAAAGCGGCTA |  | AGCGCATGTCTTCGGTTAAA |  | TCACTAAAGCGTTTTCTACC |  |  |
| HHV-6 | CCAGTCAGACAGTTGTTTCGG |  | GGCCGCATTCGTACAGATAC |  | CAGTAAGACGGGATATAATGCC |  |  |
| HHV-7 | TGGTGTCAAGCTATCCTAATGAA |  | GAGGAGAATTCTGTACCCATGG |  | CACATTTGTACTTCAAAGTAGCC |  |  |
| Necator | CTGTTTGTCGAACGGTACTTGC |  | ATAACAGCGTGCACATGTTGC |  | CTGTACTACGCATTGTATAC |  |  |
| Norovirus_GI | CGYTGGATGCGNTTYCATGA |  | CTTAGACGCCATCATCATTYAC |  | TGGACAGGAGATCGC |  |  |
| Norovirus_GII | CARGARBCNATGTTYAGRTGGATGAG |  | TCGACGCCATCTTCATTCACA |  | TGGGAGGGCGATCGCAATCT |  |  |
| O157 | TTTCACACTTATTGGATGGTCTCAA |  | CGATGAGTTTATCTGCAAGGTGAT |  | CTCTCTTTCCTCTGCGGTCCT |  |  |
| Rotarix_NSP2 | CTAACCATGCGGATAGAGTGTTC |  | TTGAAGAC GT AAATGCA TAC CAA TTC | | TCCAATAGATTGAAGTCAGTAACG |  |  |
| Rotateq_VP6 | GCGGCGTTATTTCCAAATGCACAG |  | CGTCGGCAA GCAC TGATTCA CAAA |  | ATCACGCAACAGTAGGACTCACGCTT |  |  |
| RotaVac_G9 | CGATATCGCTTCATTTTCAATC |  | AAAATCAAATCAGCTAGTTCAGACA |  | CGTTGTGTTAGTGAAATATAATTCA |  | Unpublished |
| Rotavirus | ACCATCTWCACRTRACCCTCTATGAG |  | GGTCACATAACGCCCCTATAGC |  | AGTTAAAAGCTAACACTGTCAAA |  |  |
| Salmonella | CTCACCAGGAGATTACAACATGG |  | AGCTCAGACCAAAAGTGACCATC |  | CACCGACGGCGAGACCGACTTT |  |  |
| Sapovirus | GAYCASGCTCTCGCYACCTAC | TTGGCCCTCGCCACCTAC | CCCTCCATYTCAAACACTA |  | CCRCCTATRAACCA |  |  |
| Shigella_EIEC | CCTTTTCCGCGTTCCTTGA |  | CGGAATCCGGAGGTATTGC |  | CGCCTTTCCGATACCGTCTCTGCA |  |  |
| STEC_stx1 | ACTTCTCGACTGCAAAGACGTATG |  | ACAAATTATCCCCTGWGCCACTATC |  | CTCTGCAATAGGTACTCCA |  |  |
| STEC_stx2 | CCACATCGGTGTCTGTTATTAACC |  | GGTCAAAACGCGCCTGATAG |  | TTGCTGTGGATATACGAGG |  |  |
| Strongyloides | TCCAGAAAAGTCTTCACTCTCCAG |  | TGCGTTAGAATTTAGATATTATTGTTGCT | | TCAGCTCCAGTTGAACAACAGCCTCCAA |  |  |
| Trichuris | TTGAAACGACTTGCTCATCAACTT |  | CTGATTCTCCGTTAACCGTTGTC |  | CGATGGTACGCTACGTGCTTACCATGG |  |  |
| Yersinia | TGATTCACCAGCAGCAATAC |  | GGCATCATGAAAGGCGG |  | TGTCGGTTTCTCCTTCCAGG |  |  |
