# Supplementary material for: Infectious etiology of intussusception in Indian children less than 2 years old: a matched case-control analysis
Source: Gut Pathog. 2024 Oct 23;16:61. doi: 10.1186/s13099-024-00659-z (PMC11515542; doi:10.1186/s13099-024-00659-z)
Supplement: Supplementary file 3 — Supplementary Material 3 [file 13099_2024_659_MOESM3_ESM.docx]

|  | **Cases (272)** | **Controls (272)** |
| --- | --- | --- |
| **Demographic characteristics** | | |
| Mean age in months | 8.73 | 8.9 |
| Sex (Males %) | 69.5% | 69.5% |
| **Clinical features** | | |
| Vomiting | 204 (75%) | 77 (28.31%) |
| Fever | 89 (32.72%) | 195 (71.69%) |
| Diarrhea | 112 (41.18%) | 16 (5.88%) |
| **Mode of management of intussusception** | |  |
| Hydrostatic/pneumatic reduction | 81 (29.78%) | - |
| Surgical reduction | 161 (59.19%) |  |
| Intestinal resection | 30 (11.03%) |  |
