# Supplementary material for: Infectious etiology of intussusception in Indian children less than 2 years old: a matched case-control analysis
Source: Gut Pathog. 2024 Oct 23;16:61. doi: 10.1186/s13099-024-00659-z (PMC11515542; doi:10.1186/s13099-024-00659-z)
Supplement: Supplementary file 4 — Supplementary Material 4 [file 13099_2024_659_MOESM4_ESM.docx]

|  | **OR** | **95% CI** | | **P>\|z\|** |
| --- | --- | --- | --- | --- |
| Adenovirus_C | 2.46 | 1.36 | 5.04 | 0.015 |
| Adenovirus_F | 1.37 | 0.68 | 2.77 | 0.38 |
| Astrovirus | 0.29 | 0.079 | 1.05 | 0.06 |
| CMV | 0.75 | 0.27 | 2.09 | 0.58 |
| EBV | 0.11 | 0.001 | 10.62 | 0.343 |
| Enterovirus | 1.76 | 0.93 | 3.3 | 0.08 |
| HHV6 | 2.7 | 0.19 | 37.31 | 0.458 |
| HHV7 | 11.86 | 0.34 | 417.46 | 0.173 |
| NorovirusGI | 1.24 | 0.22 | 7.15 | 0.807 |
| NorovirusGII | 0.53 | 0.27 | 1.03 | 0.061 |
| Rotavirus | 0.38 | 0.15 | 0.93 | 0.035 |
| Sapovirus | 0.9 | 0.43 | 1.92 | 0.793 |
| Cdifficile | 0.09 | 0.02 | 0.33 | <0.001 |
| EAEC | 1.03 | 0.59 | 1.77 | 0.919 |
| Shigella_EIEC | 0.85 | 0.2 | 3.57 | 0.822 |
| EPEC | 0.75 | 0.26 | 2.12 | 0.583 |
| ETEC | 0.4 | 0.11 | 1.4 | 0.151 |
| Cryptosporidium | 0.059 | 0.005 | 0.59 | 0.016 |
| Giardia | 1.71 | 0.62 | 4.73 | 0.302 |
