# Supplementary material for: Infectious etiology of intussusception in Indian children less than 2 years old: a matched case-control analysis
Source: Gut Pathog. 2024 Oct 23;16:61. doi: 10.1186/s13099-024-00659-z (PMC11515542; doi:10.1186/s13099-024-00659-z)
Supplement: Supplementary file 5 — Supplementary Material 5 [file 13099_2024_659_MOESM5_ESM.docx]

|  | **OR** | **95% CI** |  | **P>\|z\|** |
| --- | --- | --- | --- | --- |
| Adenovirus_C | 6.91 | 1.56 | 30.68 | 0.011 |
| Adenovirus_F | 1.38 | 0.41 | 4.67 | 0.607 |
| Adenovirus_pan | 1.95 | 1.01 | 3.76 | 0.046 |
| Astrovirus | 0.17 | 0.033 | 0.9 | 0.037 |
| EBV | 0.37 | 0.02 | 5.68 | 0.473 |
| Enterovirus | 0.68 | 0.22 | 2.11 | 0.499 |
| HHV7 | 15.13 | 0.73 | 314.28 | 0.079 |
| NorovirusGII | 0.55 | 0.24 | 1.27 | 0.161 |
| Sapovirus | 0.81 | 0.26 | 2.49 | 0.708 |
| C difficile | 0.04 | 0.006 | 0.18 | <0.001 |
| EAEC | 1.25 | 0.66 | 2.38 | 0.487 |
| Shigella_EIEC | 0.59 | 0.09 | 3.77 | 0.583 |
| EPEC | 0.68 | 0.2 | 2.66 | 0.529 |
| ETEC | 0.54 | 0.12 | 2.49 | 0.433 |
| Giardia | 1.18 | 0.31 | 4.52 | 0.812 |
