# Supplementary material for: Infectious etiology of intussusception in Indian children less than 2 years old: a matched case-control analysis
Source: Gut Pathog. 2024 Oct 23;16:61. doi: 10.1186/s13099-024-00659-z (PMC11515542; doi:10.1186/s13099-024-00659-z)
Supplement: Supplementary file 6 — Supplementary Material 6 [file 13099_2024_659_MOESM6_ESM.docx]

| Enteropathogen Positivity among IS cases and controls within 21 days of Rotavirus vaccine receipt | | |
| --- | --- | --- |
|  | **Positivity % among cases(N=18)** | **Positivity among controls (N=21)** |
| Adenovirus_C | 10 (55.6%) | 5 (23.8%) |
| Adenovirus_F | 11 (61.1%) | 11 (52.4%) |
| Astro | 9 (50.0% | 4 (19.0%) |
| C.diff | 0.0 | 5 (23.8%) |
| CMV | 6 (33.3%) | 4 (19.0%) |
| *Cryptosporidium* spp | 0.0 | 2 (9.5%) |
| EAEC | 6 (33.3%) | 4 (19.0%) |
| EPEC | 4 (22.2%) | 0.0 |
| EBV | 1 (5.6%) | 2 (9.5%) |
| Enterovirus | 8 (44.4%) | 7 (33.3%) |
| Giardia | 0.0 | 2 (9.5%) |
| HHV7 | 1 (5.6%) | 1 (4.8%) |
| NorovirusGII | 1 (5.6%) | 5 (23.8%) |
| Rotavirus | 3 (16.7%) | 3 (14.3%) |
| Sapovirus | 5 (27.8%) | 3 (14.3%) |
| *Shigella* spp | 0.0 | 1 (4.8%) |
